# Supplementary material for: Conditional chemoconnectomics (cCCTomics) as a strategy for efficient and conditional targeting of chemical transmission
Source: eLife. 2024 Apr 30;12:RP91927. doi: 10.7554/eLife.91927 (PMC11060718; doi:10.7554/eLife.91927)
Supplement: Figure 3—source data 3. [file elife-91927-fig3-data3.zip › Figure 3 source data3 part 1.zip!/Figure 3-Source data 3-part1/1081-1-CRISPResso_Report_8zosk6yj.zip!/CRISPResso_on_1081_3/9.Alleles_frequency_table_around_sgRNA_tttctggactacagtcccca.pdf]

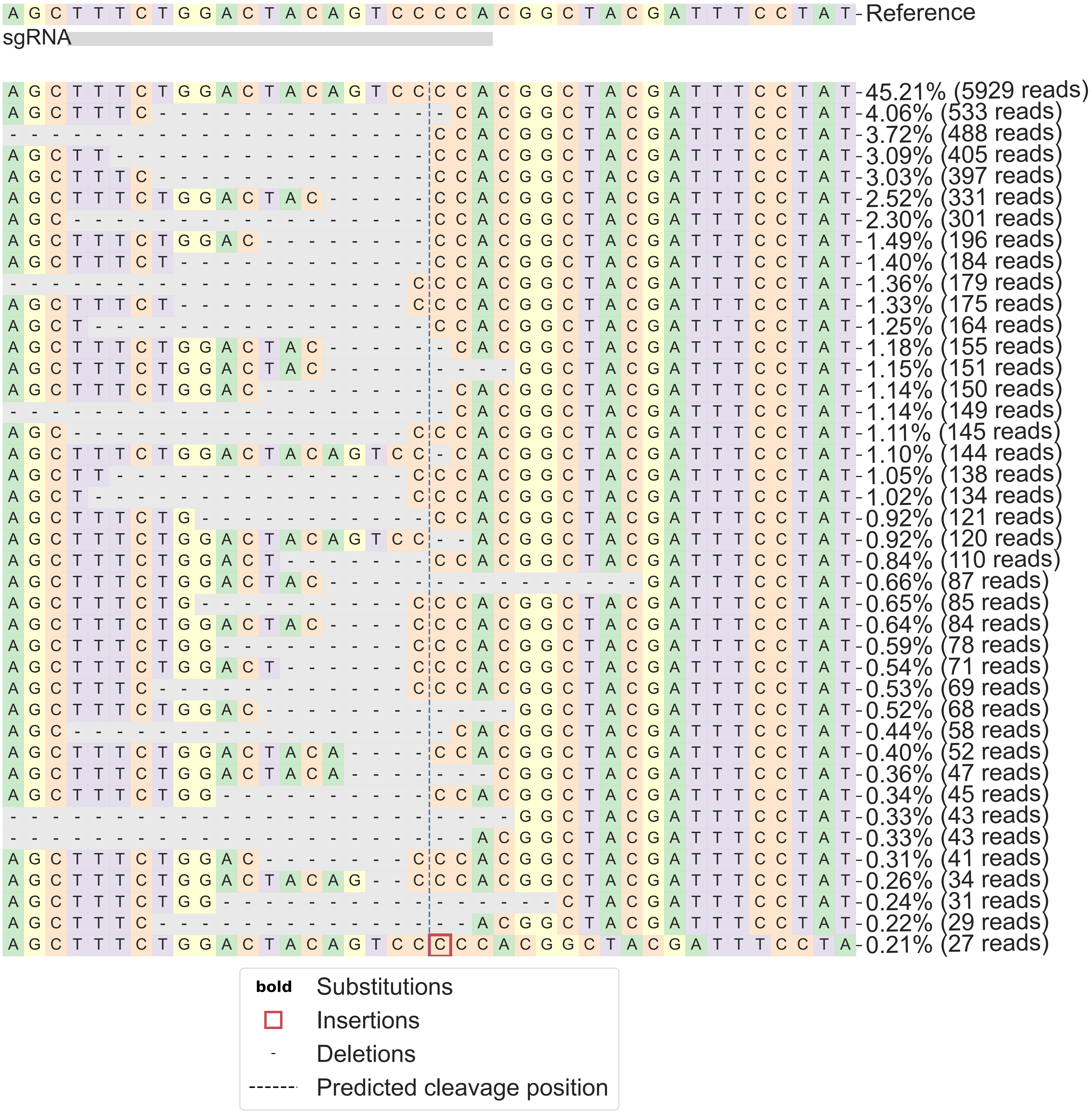

A G C T T T C T G G A C T A C A G T C C C C A C G G C T A C G A T T T C C T A T
Reference
sgRNA
45.21% (5929 reads)
4.06% (533 reads)
3.72% (488 reads)
3.09% (405 reads)
3.03% (397 reads)
2.52% (331 reads)
2.30% (301 reads)
1.49% (196 reads)
1.40% (184 reads)
1.36% (179 reads)
1.33% (175 reads)
1.25% (164 reads)
1.18% (155 reads)
1.15% (151 reads)
1.14% (150 reads)
1.14% (149 reads)
1.11% (145 reads)
1.10% (144 reads)
1.05% (138 reads)
1.02% (134 reads)
0.92% (121 reads)
0.92% (120 reads)
0.84% (110 reads)
0.66% (87 reads)
0.65% (85 reads)
0.64% (84 reads)
0.59% (78 reads)
0.54% (71 reads)
0.53% (69 reads)
0.52% (68 reads)
0.44% (58 reads)
0.40% (52 reads)
0.36% (47 reads)
0.34% (45 reads)
0.33% (43 reads)
0.33% (43 reads)
0.31% (41 reads)
0.26% (34 reads)
0.24% (31 reads)
0.22% (29 reads)
0.21% (27 reads)
bold Substitutions
Insertions
Deletions
Predicted cleavage position
